# Supplementary material for: Spatial Distance and Temporal Attentional Focus Modulate Voluntary Action Preparation and Awareness
Source: Psychophysiology. 2026 Mar 13;63(3):e70280. doi: 10.1111/psyp.70280 (PMC12983192; doi:10.1111/psyp.70280)
Supplement: Supplementary file 1 — Figure S1: Illustration of the EMG onset detection method. Figure S2: Example EMG traces illustrating response‐proximal transient activity (Participant 2). Figure S3: EEG signal from the second participant (corresponding to the EMG signal shown in Figure S2). Figure S4: Computer‐registered keypress movement initiation times for all tasks involving keypresses. Figure S5: Movement initiation time distributions by distance. Figure S6: Movement initiation time distributions by task. Figure S7: Histograms of motor times across tasks. Figure S8: The time‐course of RP across different experimental tasks with epochs aligned on EMG‐detected movement onset and without baseline correction. Figure S9: Per‐participant mean and Δmean movement initiation time (MIT) for the baseline (Decision and Action) conditions (Near and Far trials collapsed). Figure S10: Per‐participant mean and Δmean movement initiation time (MIT) for the operant (Decision and Action) conditions (Near and Far trials collapsed). Figure S11: Per‐participant movement initiation time (MIT) for the Decision baseline task. Figure S12: Per‐participant movement initiation time (MIT) for the Decision operant task. Figure S13: Per‐participant movement initiation time (MIT) for the Action baseline task. Figure S14: Per‐participant movement initiation time (MIT) for the Action operant task. Figure S15: Per‐participant movement initiation time (MIT) for the Action‐only task. Figure S16: Validation experiment for movement initiation time. [file PSYP-63-e70280-s001.docx]

**Supplemental material for**

**Spatial distance and temporal attentional focus modulate voluntary action preparation and awareness**

Gaiqing Kong, Bastien Barlerin, Clément Desoche, Luke Miller, Francesco Pavani,

Alessandro Farnè, Marine Vernet

**This includes:**

Figure S1. Illustration of the EMG onset detection method.

Figure S2. Example EMG traces illustrating response-proximal transient activity (Participant 2).

Figure S3. EEG signal from the second participant (corresponding to the EMG signal shown in Figure S2).

Figure S4. Computer-registered keypress movement initiation times for all tasks involving keypresses.

Figure S5. Movement initiation time distributions by distance.

Figure S6. Movement initiation time distributions by task.

Figure S7. Histograms of motor times across tasks.

Figure S8. The time-course of RP across different experimental tasks with epochs aligned on EMG-detected movement onset and without baseline correction.

Figure S9. Per-participant mean and Δmean movement initiation time (MIT) for the baseline (Decision and Action) conditions (Near and Far trials collapsed).

Figure S10. Per-participant mean and Δmean movement initiation time (MIT) for the operant (Decision and Action) conditions (Near and Far trials collapsed).

Figure S11. Per-participant movement initiation time (MIT) for the Decision baseline task.

Figure S12. Per-participant movement initiation time (MIT) for the Decision operant task.

Figure S13. Per-participant movement initiation time (MIT) for the Action baseline task.

Figure S14. Per-participant movement initiation time (MIT) for the Action operant task.

Figure S15. Per-participant movement initiation time (MIT) for the Action-only task.

Figure S16. Validation experiment for movement initiation time.

**
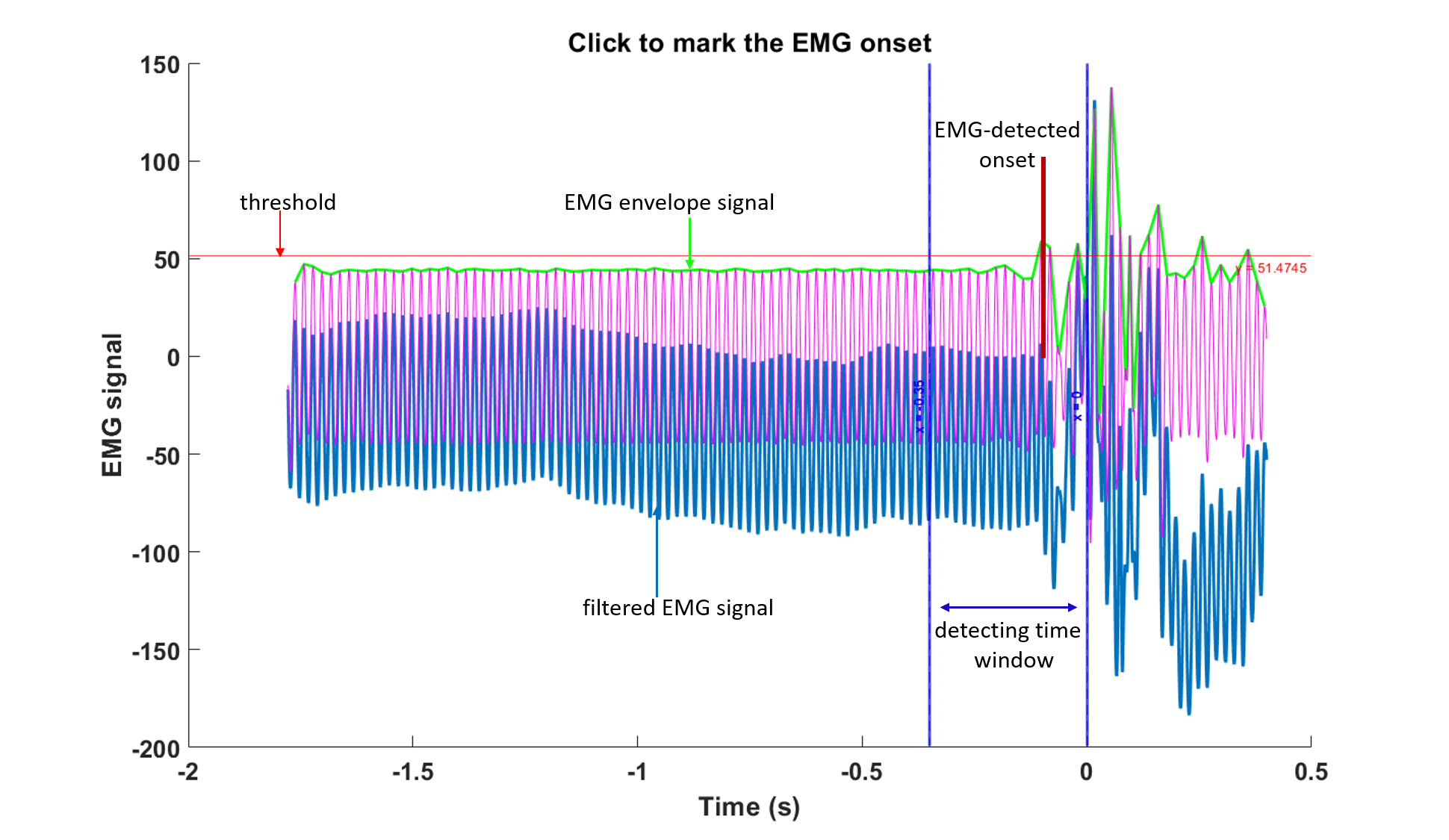
**

**Figure S1. Illustration of the EMG onset detection method.** The blue waveform represents the original EMG signal and the purple waveform the filtered EMG signal, processed to remove noise and enhance signal clarity. The EMG envelope signal (green line) is obtained by rectifying and smoothing the filtered signal, providing a more stable representation of muscle activation levels. A threshold (red horizontal line, 50% of the Peak-Baseline difference) is used to determine the onset of muscle activity. The detection period is marked by two vertical blue dashed lines at x=−0.35 and x=0 (computer-registered keypress onset), defining the time window in which EMG onset is assessed. The EMG-detected movement onset is marked by the vertical red line. If the automatically detected onset is not optimal, the user is prompted to manually select the onset point within this window for further analysis.


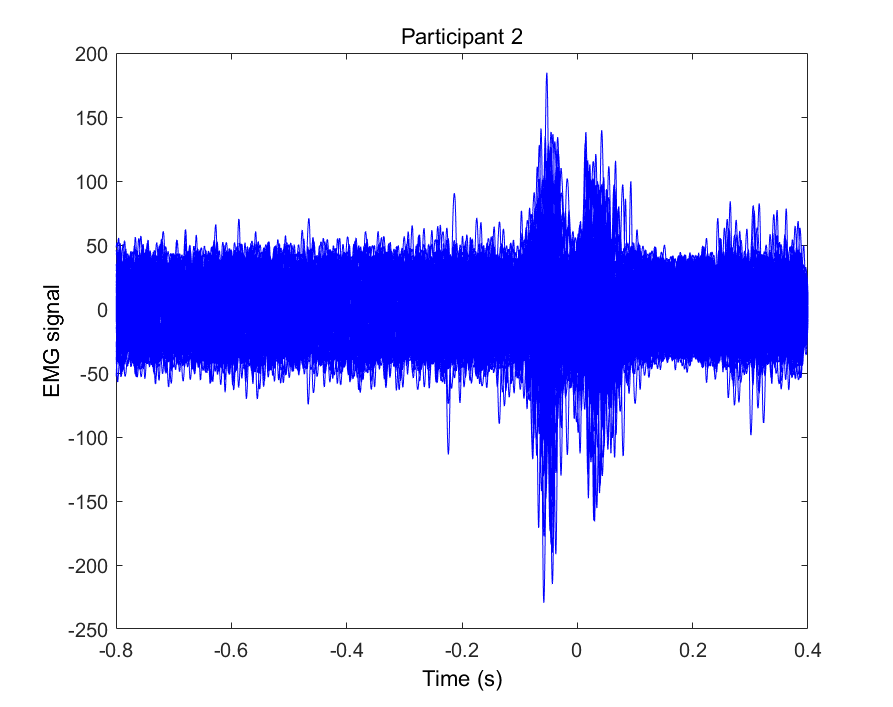


**Figure S2. Example EMG traces illustrating response-proximal transient activity (Participant 2).** Overlay of all trials from Subject 2, time-locked to the computer-registered keypress (t = 0 s). It shows a consistent, abrupt change in EMG activity immediately prior to the keypress across trials, consistent with a rapid muscle activation pattern and supporting a movement-related origin of the response-proximal transient deflections observed in the EEG.


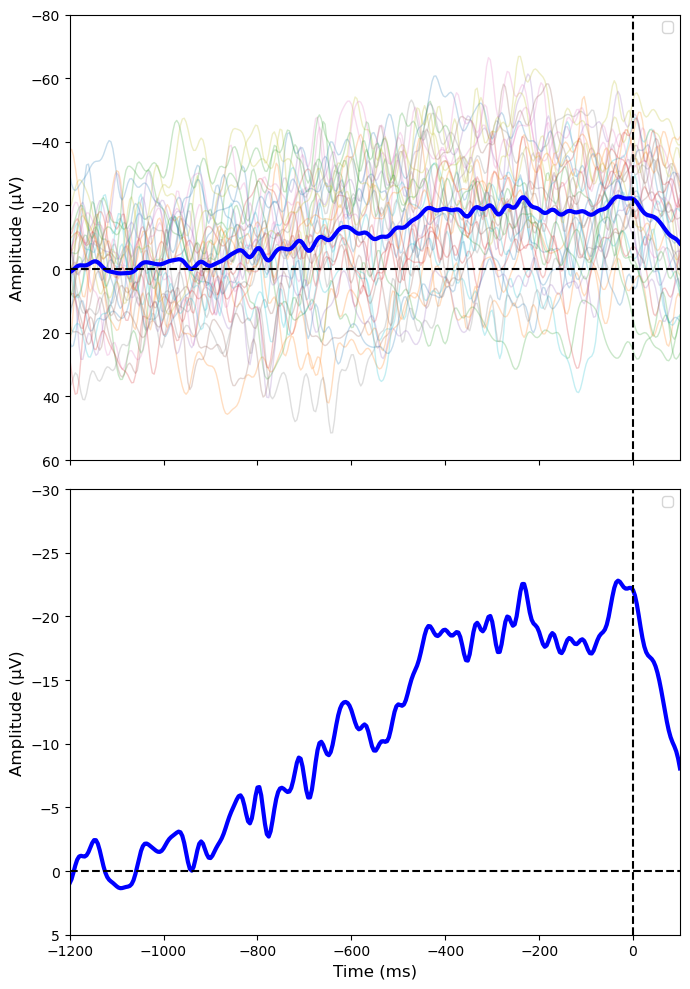


**Figure S3.** **EEG signal from the second participant (corresponding to the EMG signal shown in Figure S2).** Top panel: thin multicolored traces show individual single-trial EEG epochs; the thick blue trace shows the trial-average waveform (RP). The vertical dashed line marks movement onset (0 ms) and the horizontal dashed line marks the 0 µV reference. Bottom panel: the trial-averaged waveform only (blue), displayed with a compressed y-scale to facilitate visualization of the RP.

**Movement initiation time**

**
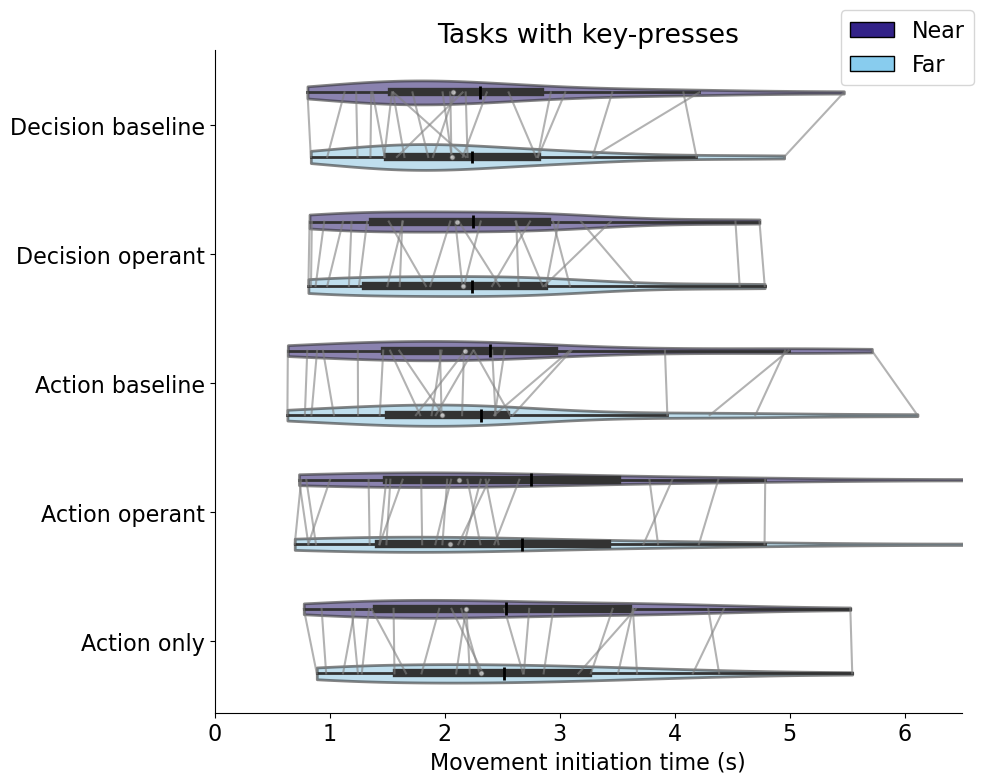
**

**Figure S4.** **Computer-registered keypress movement initiation times for all tasks involving keypresses.** The light blue and purple violins distinguish between near and far distances, respectively. Each violin's width reflects the probability density of data points across different movement initiation time values on the x-axis, offering a visual portrayal of data distribution within each task. The white dot within each violin represents the median movement initiation time, the thick horizontal line represents the interquartile range, and the vertical black bar represents the mean movement initiation time within each task.

For the computer-registered keypress movement initiation time (**Fig. S2**), the paired t-test for the Action-only task showed no significant difference between the clock presented near (Mean ± SE = 2.51 ± 0.24 s) and far (Mean ± SE = 2.48 ± 0.23 s, t (24) = .65, p = .52). The 2 (report instruction: decision vs. action) × 2 (distance: near vs. far) × 2 (tone presence: baseline vs. operant task) three-way mixed design ANOVA showed no significant main effect of spatial distance (F (1,24) = 0.25, p = 0.62, η_p_^2^ = .01), of report instruction (F (1,21) = 0.72, p = .41, η_p_^2^ = .03), and of tone presence (F (1,24) = 0.52, p = .48, η_p_^2^ = .02) and no interaction was significant (p > 0.41). These results suggest that PPS did not significantly influence the overall timing of keypresses in all tasks. Similarly, results from EMG-detected movement onset times also showed no significant influence of PPS on movement onset timing in all tasks.

**Movement initiation time distributions by distance**


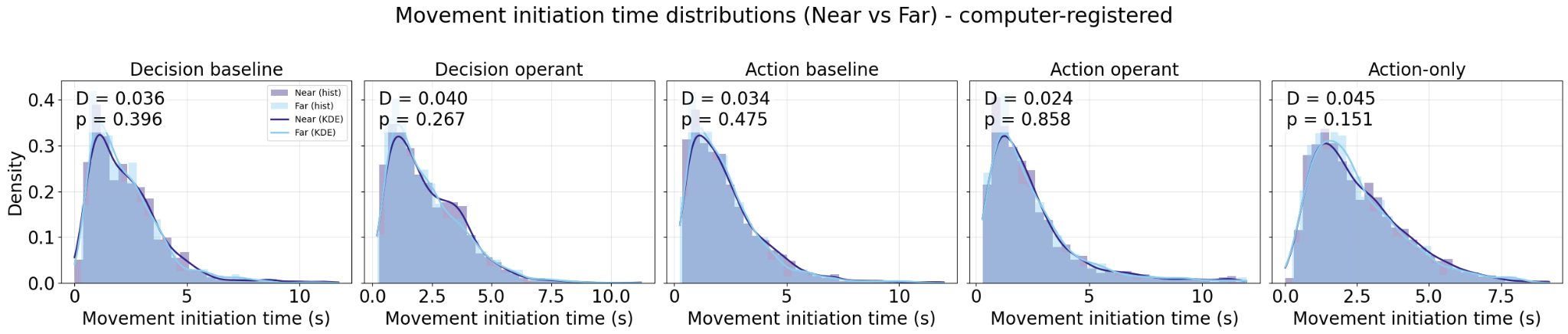


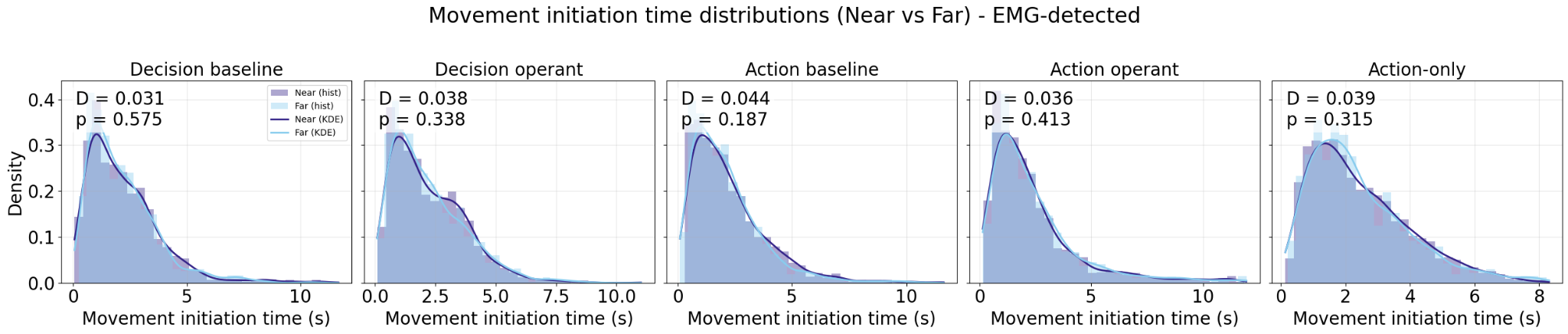


**Figure S5. Movement initiation time distributions by distance.** Distributions of movement initiation times for Near (purple) and Far (light blue) conditions for the five tasks: Decision baseline, Decision operant, Action baseline, Action operant, and Action-only. Panels show probability density estimates (kernel density functions, KDEs) overlaid on histograms of individual movement initiation times (in seconds), pooled across participants and trials. The top panel displays computer-registered movement initiation times, and the bottom panel shows EMG-detected movement initiation times. The Kolmogorov–Smirnov statistics (D, p) reported in each panel indicate that no significant differences were found between Near and Far conditions in any task (all p > 0.15).

**Movement initiation time distributions by task**


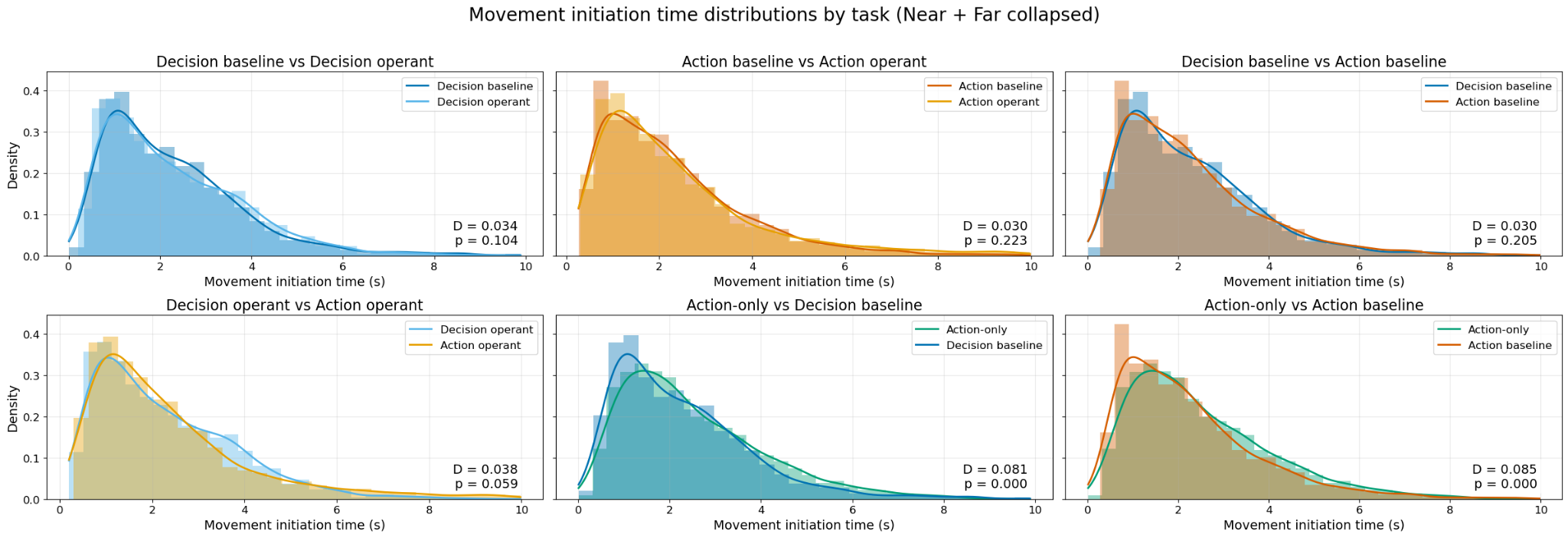


**Figure S6. Movement initiation time distributions by task.** Each subplot compares the movement initiation time (MIT) distributions between two task conditions using density histograms and KDEs. The top row shows comparisons between baseline and operant versions of the Decision task (left), the Action task (middle), and Decision vs. Action baseline tasks (right). The bottom row shows comparisons between Decision vs. Action operant tasks (left), Action-only vs. Decision baseline (middle), and Action-only vs. Action baseline (right). For each comparison, the Kolmogorov–Smirnov statistics (D, p) are reported to quantify whether the two distributions differ significantly. Distributions largely overlap across Decision and Action tasks, with no significant differences except when the Action-only condition is involved, which exhibits a distinct distribution characterized by slower and less sharply peaked initiation times.

**
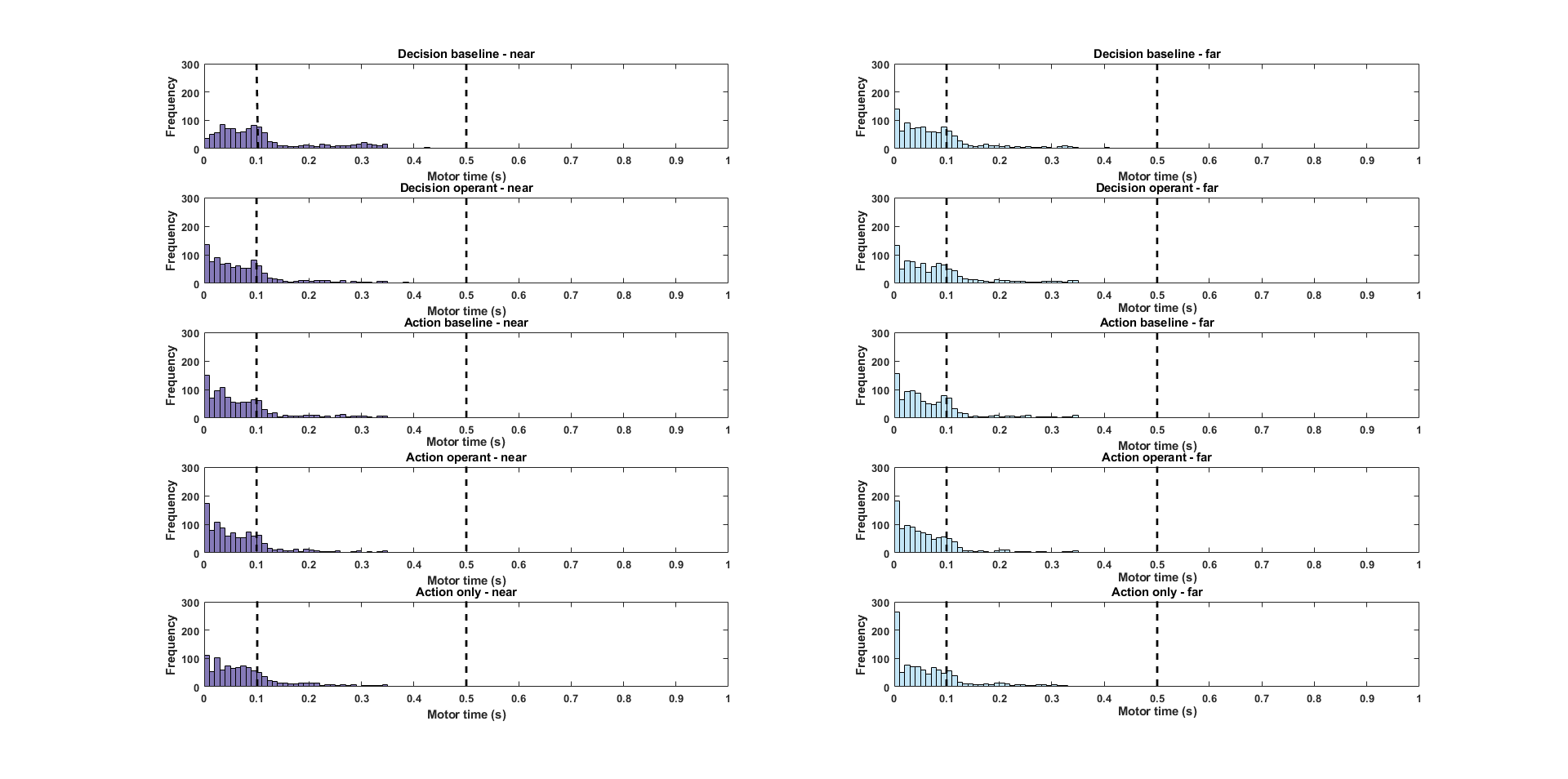
**

**Figure S7. Histograms of motor times (i.e., keypress-EMG movement initiation time (MIT) differences) across tasks.** The distribution of motor times within each task: Decision Baseline, Decision Operant, Action Baseline, Action Operant, and Action-only, with both near and far distance settings. The x-axis represents the motor time in seconds, while the y-axis represents the frequency of occurrences. The histograms show that most motor times fall within the critical range of 0 to 0.1 seconds, but an interesting second part of the distribution occurred within 0.1-0.5 s. The dashed vertical lines highlighted this critical range (0.1 to 0.5 seconds) used for further analyses (see “Movement initiation times and motor times” in the Results of the main manuscript).

**
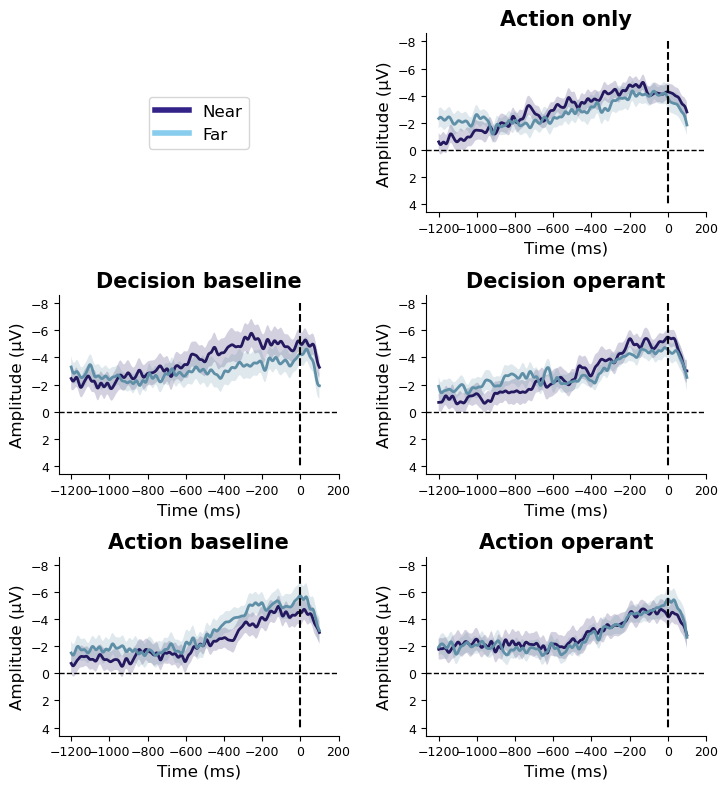
**

**Figure S8. The time-course of RP across different experimental tasks with epochs aligned on EMG-detected movement onset and without baseline correction.** The vertical dashed line at time zero represents the EMG-detected movement onset. The light blue line indicates the RP when the clock was in close proximity to the participants, while the purple line depicts the RP when the clock was positioned further away. The shaded regions surrounding each line represent standard deviation errors of the mean.

**Robustness analysis**

To directly test whether a small number of extreme participants could be driving the RP findings, we performed a robustness analysis: we identified participants with the most extreme Decision–Action timing differences (P1, P9, P20) and re-ran the 2 × 2 × 2 repeated-measures ANOVA after excluding them (n = 22). The key RP result remained the same: for the early RP mean amplitude, the critical Report × Tone interaction was still significant with an even larger effect size (F (1,21) = 11.89, p = .0024, ηp² = .361), while other effects remained non-significant. For the early RP slope, the overall pattern was also preserved (with Distance remaining significant: F (1,21) = 5.03, p = .036, ηp² = .193). These robustness checks indicate that the main RP conclusions are not driven by these participants.


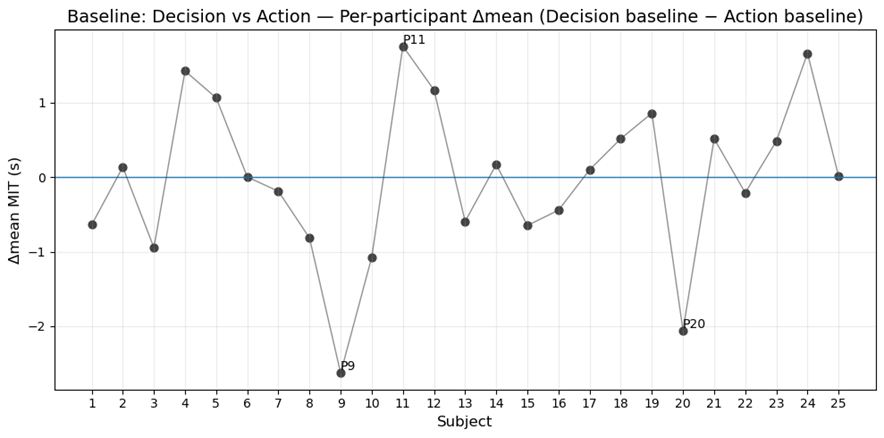

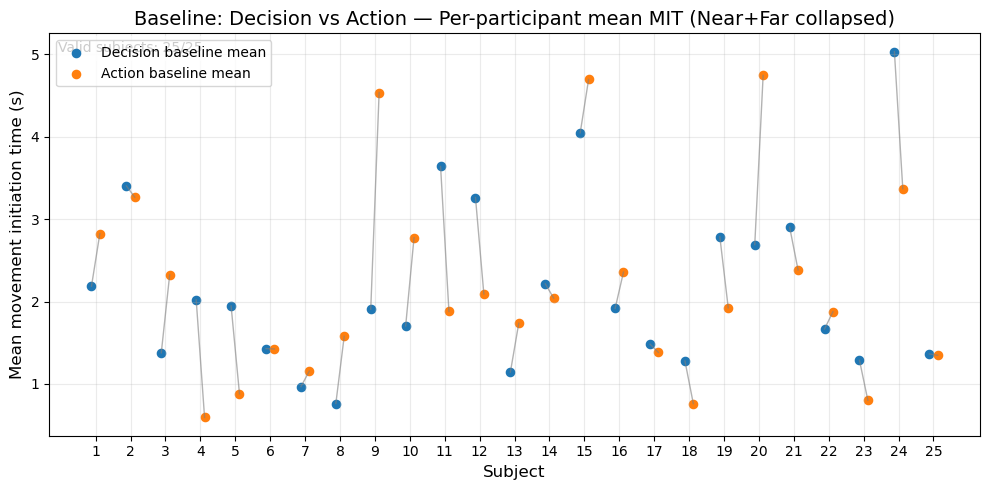


**Figure S9. Per-participant mean and Δmean movement initiation time (MIT) for the baseline (Decision and Action) conditions (Near and Far trials collapsed).** Top panel: For each participant, dots indicate the mean MIT in the Decision baseline task (blue) and the Action baseline task (orange). Grey line segments connect the two task means within each participant, illustrating within-subject task differences. Bottom panel: Per-participant difference values computed as Δmean = Decision baseline − Action baseline. Values above zero indicate slower initiation in the Decision baseline task relative to the Action baseline task; values below zero indicate faster initiation in the Decision baseline task. The horizontal line at zero marks no difference.


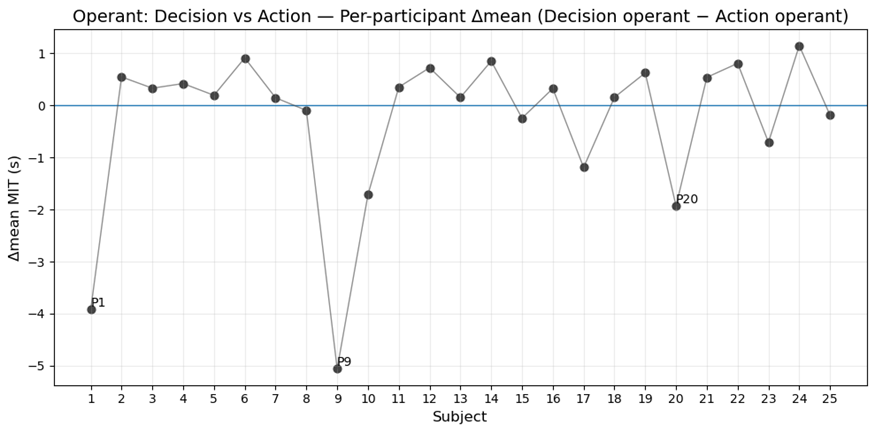

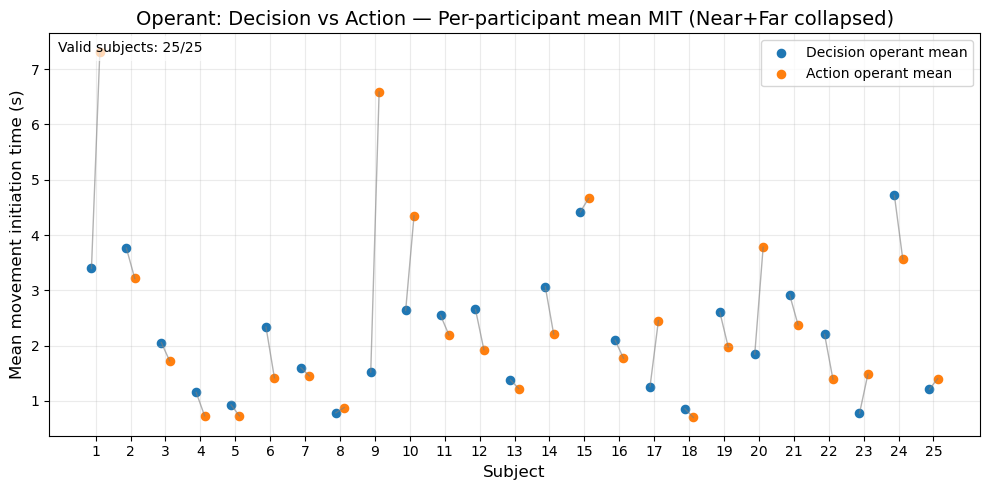


**Figure S10. Per-participant mean and Δmean movement initiation time (MIT) for the operant (Decision and Action) tasks (Near and Far trials collapsed).** Top panel: For each participant, dots indicate the mean MIT in the Decision operant task (blue) and the Action operant task (orange). Gray line segments connect the two task means within each participant, illustrating within-subject task differences. Bottom panel: Per-participant difference values computed as Δmean = Decision operant − Action operant. Values above zero indicate slower initiation in the Decision operant task relative to the Action baseline task; values below zero indicate faster initiation in the Decision operant task. The horizontal line at zero marks no difference.


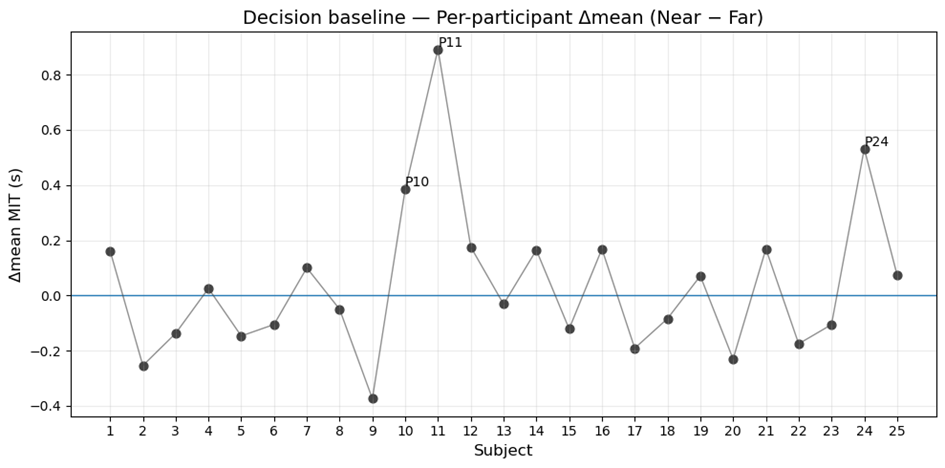

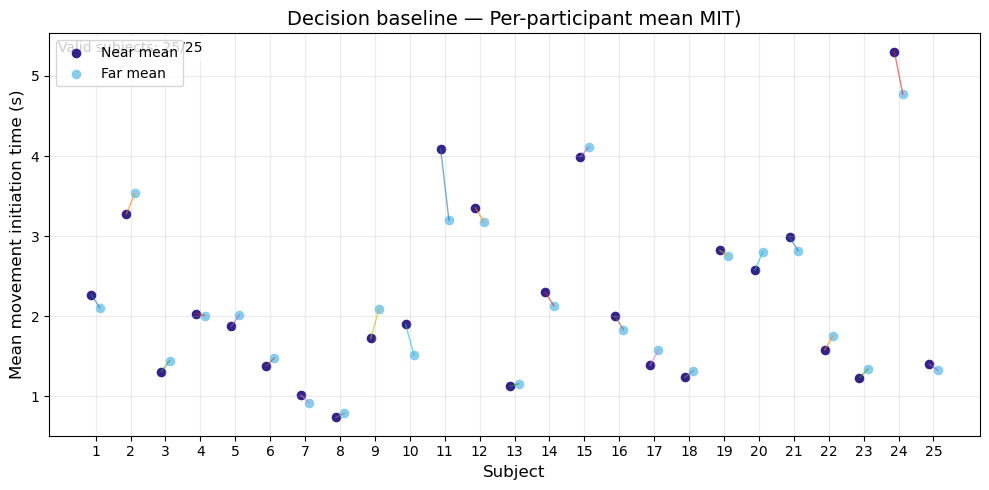


**Figure S11. Per-participant movement initiation time (MIT) for the Decision baseline task.** Top panel: For each participant, dots indicate the mean MIT in the Near condition (dark blue) and the Far condition (light blue). Gray line segments connect Near and Far means within each participant, illustrating within-subject distance differences. Bottom panel: Per-participant difference values computed as Δmean = Near − Far. Values above zero indicate slower initiation for Near relative to Far; values below zero indicate faster initiation for Near relative to Far. The horizontal line at zero marks no difference.


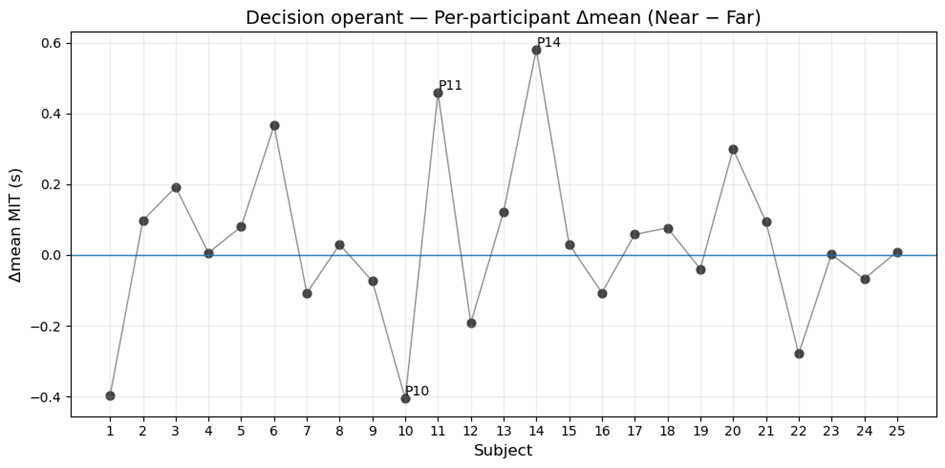

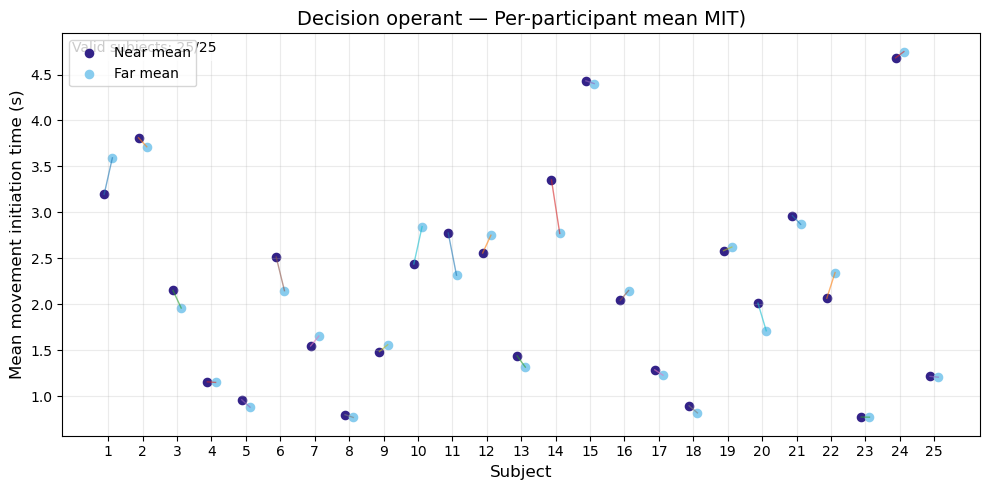


**Figure S12. Per-participant movement initiation time (MIT) for the Decision operant task.** Top panel: For each participant, dots indicate the mean MIT in the Near condition (dark blue) and the Far condition (light blue). Gray line segments connect Near and Far means within each participant, illustrating within-subject distance differences. Bottom panel: Per-participant difference values computed as Δmean = Near − Far. Values above zero indicate slower initiation for Near relative to Far; values below zero indicate faster initiation for Near relative to Far. The horizontal line at zero marks no difference.


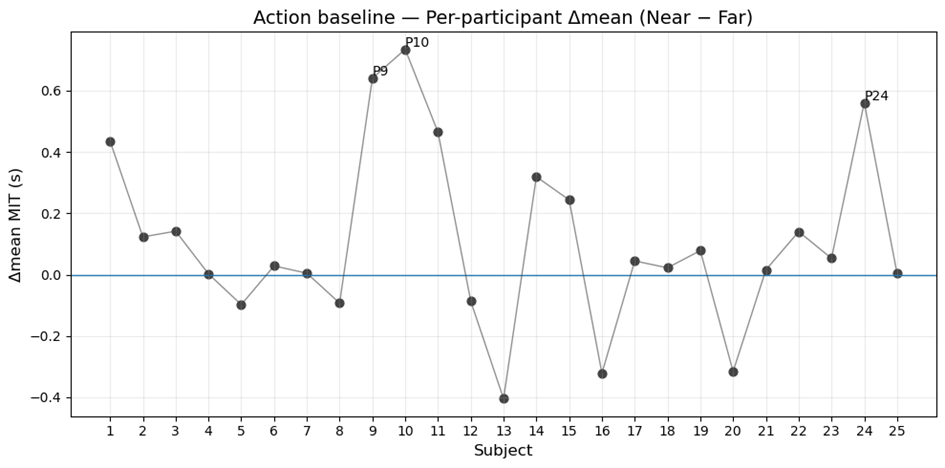

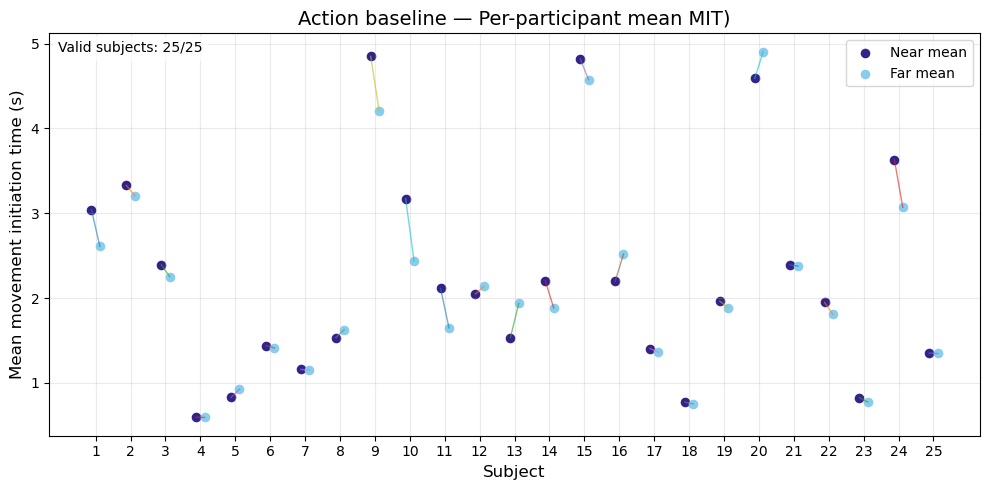


**Figure S13. Per-participant movement initiation time (MIT) for the Action baseline task.** Top panel: For each participant, dots indicate the mean MIT in the Near condition (dark blue) and the Far condition (light blue). Gray line segments connect Near and Far means within each participant, illustrating within-subject distance differences. Bottom panel: Per-participant difference values computed as Δmean = Near − Far. Values above zero indicate slower initiation for Near relative to Far; values below zero indicate faster initiation for Near relative to Far. The horizontal line at zero marks no difference.


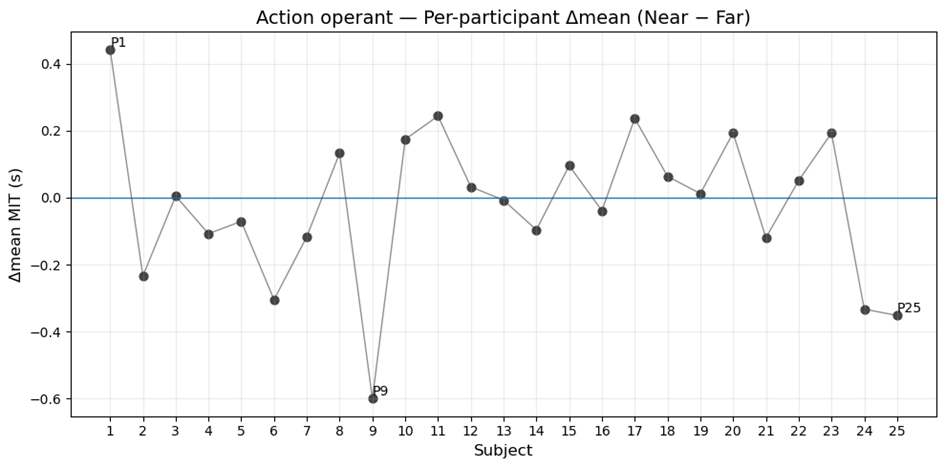

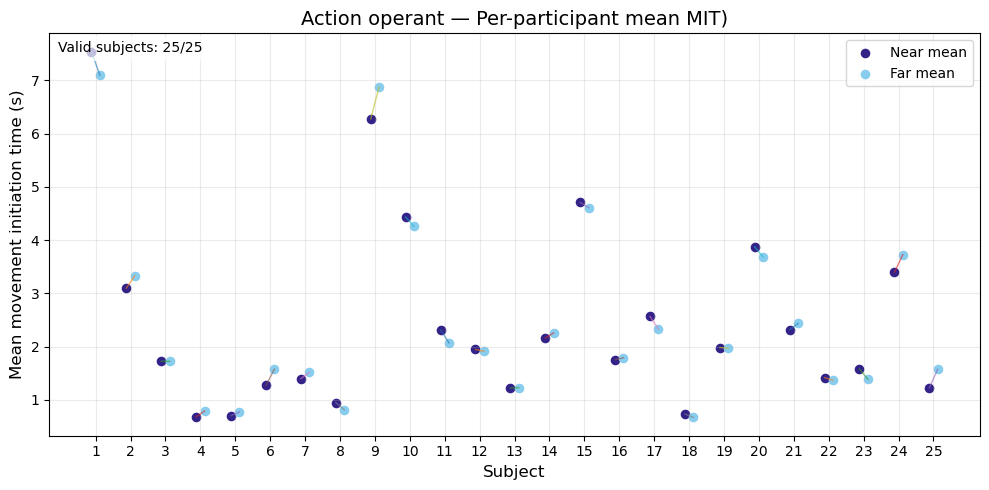


**Figure S14. Per-participant movement initiation time (MIT) for the Action operant task.** Top panel: For each participant, dots indicate the mean MIT in the Near condition (dark blue) and the Far condition (light blue). Gray line segments connect Near and Far means within each participant, illustrating within-subject distance differences. Bottom panel: Per-participant difference values computed as Δmean = Near − Far. Values above zero indicate slower initiation for Near relative to Far; values below zero indicate faster initiation for Near relative to Far. The horizontal line at zero marks no difference.


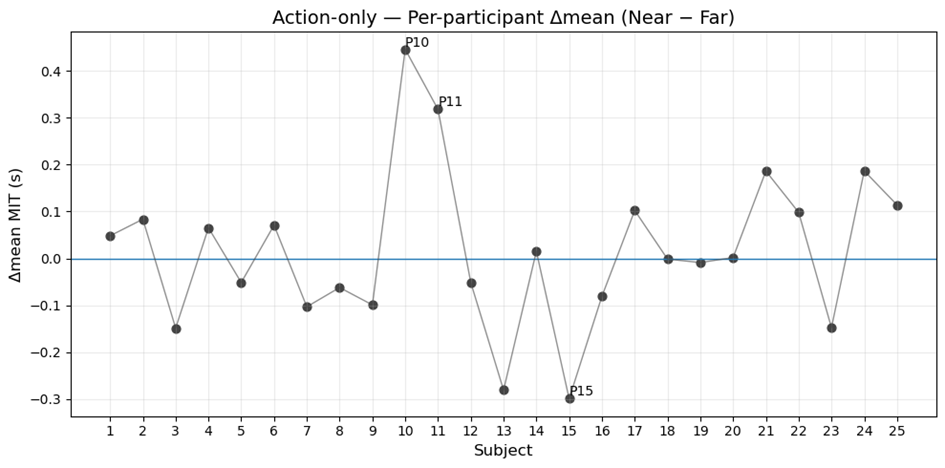

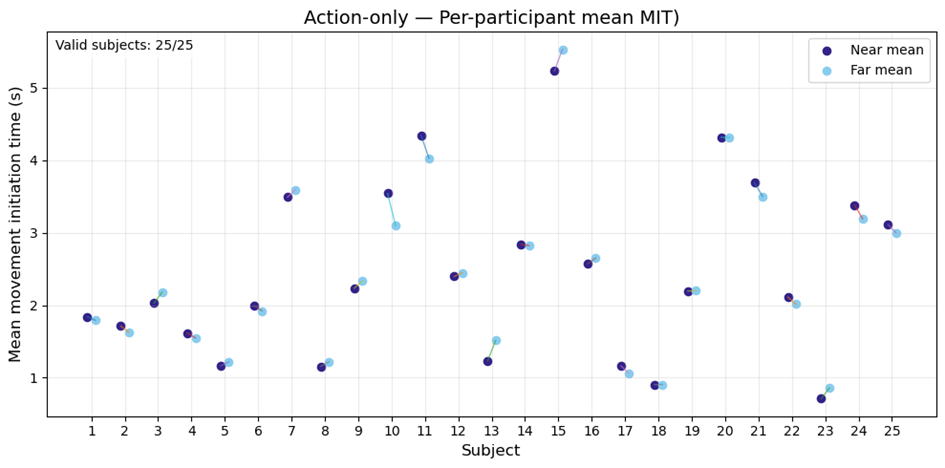


**Figure S15. Per-participant movement initiation time (MIT) for the Action-only task.** Top panel: For each participant, dots indicate the mean MIT in the Near condition (dark blue) and the Far condition (light blue). Gray line segments connect Near and Far means within each participant, illustrating within-subject distance differences. Bottom panel: Per-participant difference values computed as Δmean = Near − Far. Values above zero indicate slower initiation for Near relative to Far; values below zero indicate faster initiation for Near relative to Far. The horizontal line at zero marks no difference.


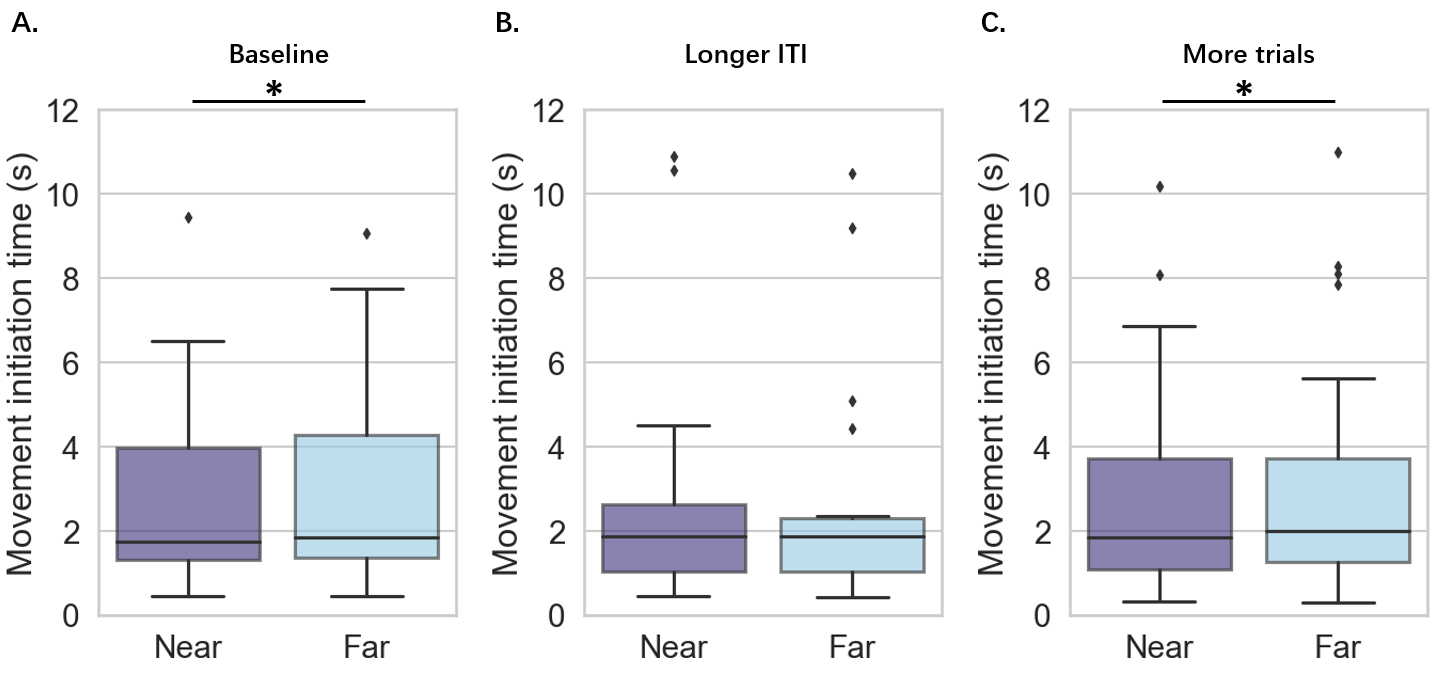


**Figure S16. Validation experiment for movement initiation time.** To determine whether and which factors—longer inter-trial intervals (ITI, **Fig. S7B**) or a higher number of repeated trials (**Fig. S7C**)—affect movement time, we conducted a follow-up validation experiment with three conditions from 20 participants (12 females, mean age = 27 years, range 20–47 years). The first condition (**Fig. S7A**) replicated the Action-only condition from our previous study (Kong et al., 2024), with shorter ITIs (randomly chosen from 1s to 2s) and fewer trials (30 trials each for near and far). The second condition (**Fig. S7B**) increased the ITI to match that of the current study (randomly chosen from 2s to 2.5s), while the third condition (**Fig. S7C**) increased the number of trials to match that of the current study (50 trials each for near and far). Thus, when comparing the first and second conditions, only one parameter differed between them. The results showed that participants initiated the action significantly earlier in the near space compared to the far space in the first and third conditions (t (19) = -2.24, p = 0.037; t (19) = -2.34, p = 0.03, respectively). However, in the second condition, where only the ITI was increased, there was no significant difference in movement time between near and far spaces (t (19) = 1.26, p = 0.22). These findings suggest that the increased ITI in the current study may have equalized the distribution of movement time in the faster range, thereby eliminating the near–far modulation. This likely explains the discrepancy in movement times observed between our previous study and the current one.
